# A comparison of methods for health policy evaluation with controlled pre‐post designs

**DOI:** 10.1111/1475-6773.13274

**Published:** 2020-02-12

**Authors:** Stephen O'Neill, Noemi Kreif, Matt Sutton, Richard Grieve

**Affiliations:** ^1^ J.E. Cairnes School of Business and Economics National University of Ireland Galway Galway Ireland; ^2^ Department of Health Services Research and Policy London School of Hygiene and Tropical Medicine London UK; ^3^ Centre for Health Economics University of York York UK; ^4^ Health Organisation, Policy and Economics School of Health Sciences University of Manchester Manchester UK; ^5^ Melbourne Institute of Applied Economic and Social Research University of Melbourne Melbourne Victoria Australia

**Keywords:** difference‐in‐differences, interactive fixed effects, pay‐for‐performance, policy evaluation, synthetic control

## Abstract

**Objective:**

To compare interactive fixed effects (IFE) and generalized synthetic control (GSC) methods to methods prevalent in health policy evaluation and re‐evaluate the impact of the hip fracture best practice tariffs introduced for hospitals in England in 2010.

**Data Sources:**

Simulations and Hospital Episode Statistics.

**Study Design:**

Best practice tariffs aimed to incentivize providers to deliver care in line with guidelines. Under the scheme, 62 providers received an additional payment for each hip fracture admission, while 49 providers did not. We estimate the impact using difference‐in‐differences (DiD), synthetic control (SC), IFE, and GSC methods. We contrast the estimation methods' performance in a Monte Carlo simulation study.

**Principal Findings:**

Unlike DiD, SC, and IFE methods, the GSC method provided reliable estimates across a range of simulation scenarios and was preferred for this case study. The introduction of best practice tariffs led to a 5.9 (confidence interval: 2.0 to 9.9) percentage point increase in the proportion of patients having surgery within 48 hours and a statistically insignificant 0.6 (confidence interval: −1.4 to 0.4) percentage point reduction in 30‐day mortality.

**Conclusions:**

The GSC approach is an attractive method for health policy evaluation. We cannot be confident that best practice tariffs were effective.

**What this study adds**

- Health policy evaluations with pre‐post designs are challenging as the parallel trends assumption underlying difference‐in‐differences estimation often does not hold for all outcomes.
- This was the case for the evaluation of the best practice tariffs (BPT) for hip fractures, a pay‐for‐performance scheme, introduced for hospitals in the English NHS.
- Alternative estimation methods have yielded contrasting estimates of the impacts of this BPT.
- In our simulations, the generalized synthetic control approach outperformed more commonly used methods (difference‐in‐differences and synthetic control methods) and hence was the preferred approach for the case study.
- It suggests that the BPT for hip fractures increased the proportion of patients who had surgery within 48 hours of admission, but did not statistically significantly reduce 30‐day mortality.

## 1 INTRODUCTION

Health policy evaluations commonly use data before and after a policy change and assume that, without the intervention, the expected outcomes for the treated and control groups would have followed parallel trends. This assumption underpins the standard difference‐in‐differences (DiD) estimator and implies that any differences between the comparator groups due to unobserved confounders are time‐constant. However, the “parallel trends” assumption is often implausible, particularly in a health policy setting. When the parallel trends assumption is violated, DiD approaches provide biased estimates of the effect of the health policy.^1^, ^2^ DiD has been widely applied to policy evaluations within health economics^4^, ^5^, ^6^, ^7^, ^8^, ^9^, ^10^ and health services research.^11^, ^12^, ^13^, ^14^, ^15^ As recently illustrated in re‐evaluating a pay‐for‐performance (P4P) scheme,^3^ a study's policy conclusions can rest on the approach taken to causal inference.^3^

The synthetic control (SC) method^16^, ^17^ has been viewed as an attractive alternative to DiD as it avoids the parallel trends assumption. In essence, the SC method constructs a comparator for the intervention group, the synthetic control, as a weighted average of the available control units. Each unit is weighted to ensure that the mean outcomes of the synthetic control track those of the treated unit(s) prior to the intervention.^3^, ^17^, ^18^, ^19^, ^20^, ^21^, ^22^, ^23^, ^24^ However, despite its wide use, critics have shown that the SC approach may provide biased estimates in settings when few pre‐intervention periods are available^2^, ^25^; treatment assignment is correlated with time‐varying unobserved confounders,^26^ or where the outcomes of the treated units cannot be obtained by weighting the control units' outcomes by values between 0 and 1 (ie, the treated units are not within the “convex hull”), leading to poor overlap. 17, 25, 27 Statistical inference is also somewhat problematic under the SC approach.^28^ Concerns about the DiD and SC approaches have encouraged recent methodological advances.^29^, ^30^, ^31^, ^32^, ^33^, ^34^, ^35^ However, these methods have not been considered in the health policy evaluation domain, which is characterized by particular challenges, notably the (im)plausibility of the parallel trends assumption, the possibility of heterogeneous treatment effects, and that there may be few pretreatment periods. Here, we consider two of these approaches: (a) interactive fixed effect (IFE) models, and (b) the generalized synthetic control (GSC) method, both are novel to this context.

IFE models are flexible regression approaches that allow for multiple time‐constant unobserved covariates, each of which may have effects that vary across time^36^, ^37^, ^38^, ^39^ relaxing the parallel trends assumption.^40^ IFE models nest the fixed effects models routinely used within DiD estimation, but may produce biased estimates when policy effects are modified by unobserved covariates, that is effects are heterogeneous.^41^ For instance, hospital quality, which is generally unobserved, may moderate the effect that a new health policy has on outcomes.

The GSC method^41^ seeks to overcome this limitation by combining insights from the SC literature with the efficiency gains of IFE models. The GSC approach allows a separate (counterfactual) potential outcome to be estimated for each treated unit, allowing heterogeneous treatment effects to be consistently estimated. It has been argued that the GSC method maintains the approximately unbiasedness property of the SC estimator but offers improved efficiency. Despite these desirable features, the GSC method has not been considered in a published health policy evaluation.^1^

We contrast the IFE and GSC methods with DiD and SC methods in a case study and in Monte Carlo simulations. We revisit an evaluation of a pay‐for‐performance scheme, best practice tariffs (BPT) for hip fractures, introduced for hospitals in the English NHS.^2^, ^42^ The incidence of hip fractures in the UK is rising annually and is currently estimated at 10.2 per 10 000 per year.^43^ The cost to the hospital services of hip fracture are substantial, and have been estimated to be £1,131 million in the year of the fracture.^44^ Thus the impact of policies such as BPT are of interest to policymakers. Our simulation study extends the precedent comparison of Xu,^41^ by considering settings relevant to the HSR context, namely few (<10) pretreatment periods, highly imbalanced numbers of treatment vs control units, and serial correlation. While all methods were susceptible to shocks that impacted treated and control units differently in the post‐treatment period, the simulations show that the IFE approach otherwise avoids bias when treatment effects are homogenous but provides biased estimates under heterogeneity. By contrast, the GSC method reports efficient estimates with low bias in the presence of nonparallel trends, heterogeneous effects, and relatively few pretreatment periods.

## 2 MOTIVATING EXAMPLE: EVALUATION OF A BEST PRACTICE TARIFFS SCHEME (BPT)

Hospital pay‐for‐performance (P4P) schemes link a portion of provider income to achieving predefined quality targets. These schemes intend to encourage providers to engage in “desirable” behaviors. However, P4P schemes may shift resources toward rewarded vs unrewarded dimensions of care quality, and so have negative spill‐over effects.^45^ A number of studies have concluded that hospital pay‐for‐performance schemes have not had the desired impact.^14^, ^46^, ^47^, ^48^, ^49^, ^50^, ^51^ The international evidence on P4P has been criticized for failing to provide reliable estimates of these schemes' relative effectiveness.^52^, ^53^, ^54^

The particular P4P scheme considered here, the BPT for hip fractures, was introduced for participating English NHS hospitals from April 2010,^2^, ^42^ who were paid a fixed sum, set at £445 in the 2010/11 financial year,^55^ for each hip fracture admission if certain conditions representing “best practice” were met.^2^ The BPT payments represented a considerable share of the total payment to providers for hip fracture care, 14% in 2011/12,^55^ so one might anticipate that providers would respond to these altered incentives to provide best practice care.

**Figure 1:**
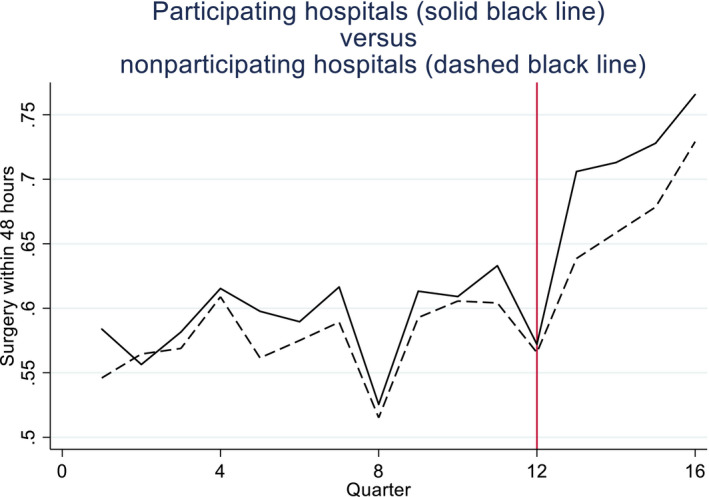
Proportions of hip fracture patients receiving surgery within 48 h of emergency admission in participating vs nonparticipating hospitals [Color figure can be viewed at http://wileyonlinelibrary.com]

**Figure 2:**
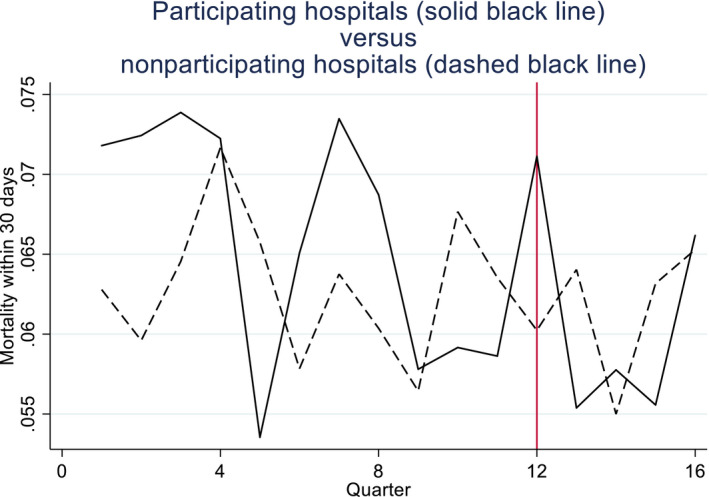
Proportions of hip fracture patients dying within 30 d of emergency admission in participating vs nonparticipating hospitals [Color figure can be viewed at http://wileyonlinelibrary.com]

A published survey and qualitative interviews suggested that BPT participation was influenced by factors unobserved by researchers^42^, ^3^, such as the resources required for this scheme, the quality of facilities available, and the expected benefits from participation. These may have had time‐varying effects on the outcomes. Hence, a priori, it was unclear whether the parallel trends assumption held for each outcome. For one outcome, the proportion of patients who had surgery within 48 hours, the parallel trends assumption appeared plausible (Figure 1), and tests suggested this assumption could not be rejected (*P* = .9255).^4^ However, for the primary outcome, mortality within 30 days, the parallel trends assumption appeared less plausible (Figure 2) and the null hypothesis of parallel trends was rejected (*P* = .039).

Previous analyses, using DiD and SC methods, found that conclusions regarding the effects of the BPT differed by method.^2^ Estimates based on DiD reported that the introduction of BPTs led to a statistically significant reduction in mortality, whereas the SC method failed to reject the null of no effect across all outcomes and indicated a smaller impact on mortality compared to DiD. However, the authors raised concerns regarding the efficiency of the SC estimates, motivating this re‐analysis using alternative methods.

We re‐analyze the data used in a previously published study,^2^ consisting of hospital admissions from 62 hospital trusts that reported receiving at least some BPT payments (treated group) and 49 trusts that reported receiving no payments under the scheme (control group). Panel data were available for twelve quarters before, and four after, the scheme's introduction. All analyses were conducted at the level of the hospital‐quarter.

The outcomes considered are the proportion of patients receiving surgery within 48 hours of an emergency admission and the proportion of patients that die within 30 days of admission. We adjust for baseline covariates according to age group, gender, and source of admission.

## 3 METHODS

Suppose there are *i* = 1,…,*n* units, and *T* time periods, where *t* = 1,…*t*' are pretreatment, and *t*' + 1,…,*T* are post‐treatment. The potential outcomes^56^ for unit *i* in period *t* in the presence and absence of treatment are denoted by $Y_{\mathrm{it}}^{1}$ and $Y_{\mathrm{it}}^{0}$, respectively. Let *D_it_* be an indicator equal to one if unit *i* is treated (exposed to the policy) in period *t* and zero otherwise. The observed outcome can be written as:

$$Y_{\mathrm{it}}=D_{\mathrm{it}}Y_{\mathrm{it}}^{1}+\left(1-D_{\mathrm{it}}\right)Y_{\mathrm{it}}^{0}$$

We assume the following factor model for the potential outcome in the absence of treatment:

$$Y_{\mathrm{it}}^{0}=X_{\mathrm{it}}^{\prime }\beta +\left(\lambda_{1t}\mu_{i1}\ldots +\lambda_{\mathrm{Rt}}\mu_{\mathrm{iR}}\right)+\varepsilon_{\mathrm{it}}$$

where $X_{\mathrm{it}}$ is a (1 × *k*) vector of observed time‐varying covariates, *β* is the (*k* × 1) vector of their coefficients, assumed to be the same for both groups, *µ_ir_* (*r* = 1, …, *R*) represents an unobserved time‐invariant variable with *λ_rt_* capturing the effect of that unobserved variable in period *t,* and $\varepsilon_{\mathrm{it}}$ represents exogenous, unobserved idiosyncratic shocks. Allowing for an additive treatment effect that may differ by individual and period (*τ_it_*), and letting ***μ_i_*** = [*μ_i_*_1_,…, *μ_iR_*] and *λ_t_* = [*λ*_1_ *_t_*,…, *λ_Rt_*], the observed outcome can be written as:

(1) $$Y_{\mathrm{it}}={X^{\prime }}_{\mathrm{it}}\beta +\lambda_{t}^{\prime }\mu_{i}+D_{\mathrm{it}}\tau_{\mathrm{it}}+\varepsilon_{\mathrm{it}}$$

The estimand of interest is the average treatment effect for the treated (ATT) after controlling for covariates, E(*τ_it_*|*D_it_* = 1, *X_it_*) over the post‐treatment period, *t* > *t*′.

### 3.1 Difference in Differences (DiD)

Note that if $\mu_{i}=\left[1,\mu_{i}\right]$ and $\lambda_{t}=\left[\lambda_{t},1\right]$, equation 1 would correspond to a two‐way fixed effects model:

(2) $$Y_{\mathrm{it}}={X^{\prime }}_{\mathrm{it}}\beta +\mu_{i}+\lambda_{t}+D_{\mathrm{it}}\tau_{\mathrm{it}}+\varepsilon_{\mathrm{it}}$$

In this case, the parallel trends assumption will hold^57^, ^58^:

$$E\left(Y_{\mathrm{it}}^{0}-Y_{it^{\prime }}^{0}|D_{\mathrm{it}}=1,X_{\mathrm{it}}\right)=E\left(Y_{\mathrm{it}}^{0}-Y_{it^{\prime }}^{0}|D_{\mathrm{it}}=0,X_{\mathrm{it}}\right)\forall t>t^{\prime }\;\left(\text{A1}:\;\text{Parallel trends}\right).$$

where *t*′ represents the final pretreatment period, and the conditional ATT can be estimated using DiD with two‐way fixed effects regression.^24^, ^59^, ^60^, ^61^, ^5^, ^6^

### 3.2 Interactive fixed effects

Interactive fixed effects models rely on an alternative set of estimation approaches for the common factor structure $\lambda_{t}{{}^{\prime }\mu }_{i}$.^37^ Here, we estimate the IFE model using the iterative principal component estimator.^37^ This approach consists of iterating between (a) estimating $\lambda_{t}$ and $\mu_{i}$ using principal components while holding $\hat{\beta }$ constant, and (b) estimating β by regressing $\left(Y-{\hat{\lambda }}_{t}^{\prime }{\hat{\mu }}_{i}\right)$ on **X**, until convergence is achieved. The number of factors to include can be chosen according to cross‐validation as described in Algorithm 1 in Xu.^41^ It is preferable to include too many rather than too few factors.^62^

One limitation of the IFE approach is that when treatment effects are moderated by the unobserved factors, the estimated average treatment effect may be biased, since the heterogeneity in treatment effects leads to biased estimates of the common factors and hence the implied treatment‐free potential outcome.

### 3.3 Synthetic control (SC) method

The synthetic control method has been shown to provide an approximately unbiased estimator of the ATT for a treated unit^17^ when outcomes are determined by a linear factor model with time‐invariant covariates (*Z_i_*), such as:

(3) $$Y_{\mathrm{it}}=\theta_{t}Z_{i}^{}+\lambda_{t}{{}^{\prime }\mu }_{i}+D_{\mathrm{it}}\tau_{\mathrm{it}}+\varepsilon_{\mathrm{it}}$$

The SC method aims to estimate the unit level causal effect *τ_it_* for the treated unit, by constructing a “synthetic control,” or a weighted average of the control units that has similar outcomes and observed covariates to the treated unit over the pre‐intervention period:

$$\sum_{j\in Control}w_{j}Y_{\mathrm{jt}}\approx Y_{1t},\forall t\leq T_{0}\;\text{and}\;\sum_{j\in Control}w_{j}Z_{i}^{}\approx Z_{1}^{},\forall t\leq T_{0}$$

where $w_{j}$ is an element of **W** representing the weight for control *j*, with $0\leq w_{j}\leq 1$. The synthetic control is formed by finding the vector of weights **W** that minimizes ${\left(X_{1}-X_{0}W\right)}^{\prime }V\left(X_{1}-X_{0}W\right)$ subject to the weights in *W* being positive and summing to 1, where X_1_ and X_0_ contain the pretreatment outcomes and covariates for the treated unit and control units, respectively,^17^ and ***V*** captures the relative importance of these variables as predictors of the outcome of interest. When X_1_ and X_0_ include all of the pre‐intervention outcomes, other covariates do not influence the weights and hence can be excluded as is done in our analysis below. If the synthetic control and treated unit have similar outcomes over an extended pre‐intervention period, it is plausible that they have similar observed and unobserved predictors of the outcome.^25^ Hence, the postintervention outcome for the synthetic control represents the counterfactual treatment‐free potential outcome for the treated unit (${\hat{Y}}_{1t}^{0}$). The SC method assumes conditional independence^2^/ignorability^74^:

$$Y_{\mathrm{it}}^{0}⊥D_{\mathrm{it}}|\left(Y_{\mathrm{ih}}^{0}\right)\;\left(\text{A2}:\;\text{Independence conditional on past outcomes}\right).$$

where $Y_{\mathrm{ih}}^{0}$ is a vector of potential outcomes in the *h* time periods prior to treatment.

Since the weights are restricted to be between 0 and 1, the treated unit must lie within the “convex hull” of the control units to avoid bias.^17^ The treatment effect for the treated unit (*i* = 1), *τ*_1_ *_t_*, can be estimated by $\left(Y_{1t}^{\prime }-{\hat{Y}}_{1t}^{0}\right)$ for each postintervention period separately, and these can be averaged over time to obtain an ATT over the postintervention period.

The SC approach can be applied to multiple treated units by applying the method to each treated unit or, as we do here, averaging across the sample of treated units to obtain a single treated unit.^18^, ^20^

### 3.4 Generalized synthetic control (GSC) method

The GSC approach^41^ assumes that treatment assignment is independent of potential outcomes conditional on the observed covariates, and *R* orthogonal, unobserved latent factors ($\lambda_{t}=\lambda_{t1},\ldots ,\lambda_{\mathrm{tR}}$) and their factor loadings ($\mu_{i}=\mu_{i1},\ldots ,\mu_{\mathrm{iR}}$)^41^:

$$\left\{Y_{\mathrm{it}}^{1},Y_{it^{}}^{0}\right\}⊥D_{i}|X_{\mathrm{it}},\lambda_{t},\mu_{i}$$

which implies that

(A3) $$E\left(Y_{\mathrm{it}}^{1},Y_{\mathrm{it}}^{0}|D_{i}=1,X_{\mathrm{it}},\lambda_{t},\mu_{i}\right)=E\left(Y_{\mathrm{it}}^{1},Y_{\mathrm{it}}^{0}|D_{i}=0,X_{\mathrm{it}},\lambda_{t},\mu_{i}\right)$$

This will hold true if the same IFE data generating process, such as equation 1 above, underlies outcomes for the treated and the control units. The key difficulty in estimating the unobserved treatment‐free potential outcome of the treated units in the post‐treatment periods is estimating $\lambda_{t}$ for the post‐treatment period and $\mu_{i}$ for each treated unit. The GSC approach tackles these difficulties as follows:^7^

First, an IFE model, $Y_{\mathrm{it}}^{0}=X_{\mathrm{it}}\beta +\lambda_{t}\mu_{i}+\varepsilon_{\mathrm{it}}$, is estimated for the control units only, for the entire sample period, yielding estimates ($\hat{\beta },{\hat{\lambda }}_{t}$) for the control units. Since $\tau_{\mathrm{it}}D_{\mathrm{it}}$ is zero in equation 1 for the control units, ($\hat{\beta },{\hat{\lambda }}_{t}$) are consistent estimates of ($\beta ,\lambda_{t}$), which are assumed to be the same for the treated and control units. If we knew $\mu_{i}$ for the treated units, we could use our estimates from the control group ($\hat{\beta },{\hat{\lambda }}_{t}$) to predict the post‐treatment treatment‐free potential outcome for the treated unit using:

(4) $${\hat{Y}}_{\mathrm{it}}^{\prime }=X_{\mathrm{it}}\hat{\beta }+{\hat{\lambda }}_{t}\mu_{i}$$

Since we do not know $\mu_{i}$ for each treated unit, the GSC method finds the value, ${\hat{\mu }}_{i}$, that minimizes the pretreatment discrepancy between the observed outcome and the predicted outcome for a given treated unit, based on [4].^41^ Using the estimates for $\hat{\beta }$ and ${\hat{\lambda }}_{t}$ from the control units and the resulting prediction ${\hat{\mu }}_{i}$ for the treated unit, we can estimate the treatment‐free potential outcome for the treated units as:

(5) $${\hat{Y}}_{\mathrm{it}}^{0}=X_{\mathrm{it}}\hat{\beta }+{\hat{\lambda }}_{t}{\hat{\mu }}_{i}$$

The estimated treatment‐free potential outcomes after the program starts can be compared to the actual outcomes for the treated units to obtain an estimated treatment effect ${\hat{\tau }}_{\mathrm{it}}=\left(Y_{\mathrm{it}}^{\prime }-{\hat{Y}}_{\mathrm{it}}^{0}\right)$ for each unit in each period. Since, unlike the IFE approach, estimates of $\hat{\beta }$, ${\hat{\lambda }}_{t}$ and ${\hat{\mu }}_{i}$ do not depend on post‐treatment information for the treated units, ${\hat{\tau }}_{\mathrm{it}}$ is not biased by heterogeneous treatment effects.

As with the SC method, when the number of pretreatment periods is small, it becomes harder to distinguish between $\mu_{i}$ and $\varepsilon_{\mathrm{it}}$, which can lead to biased estimates of the treatment effect. This bias shrinks to zero as both the number of pretreatment periods and the size of the control group grow.^41^ Unlike the SC method, the GSC method conveniently allows for time‐varying observed covariates. The GSC approach requires data be available for *R + 1* pre‐intervention periods.^8^

## 4 IMPLEMENTING THE METHODS IN THE RE‐ANALYSIS OF BPT FOR HIP FRACTURES

We replicated the DiD and SC estimations reported in a previously published study.^2^ The DiD estimation was undertaken at the hospital‐level and controlled for covariates (age, gender, source of admission), together with two‐way fixed effects for time periods and hospitals. The SC method averaged the treated units to define a single treated unit, and a synthetic control was formed from the control units. In our implementation of the SC method, we included all of the pre‐intervention outcomes as separate variables in the *X*_0_ and *X*_1_ matrices. The variable weights were determined simultaneously with the synthetic control weights^17^ as implemented in the Stata package *synth*.

The IFE model was estimated using the iterative principal component estimator.^37^ In our implementations of IFE and GSC, we included the time‐varying covariates in the IFE model, two‐way fixed effects, and up to five interactive fixed effects with the number chosen by cross‐validation, following Algorithm 1 in Xu.^41^ For inference, we used a parametric bootstrap with 500 replications.

For each method, we report *p*‐values using the most common approach to inference for each approach, but recognizing that there are differences across methods that limit comparability of the resultant *p*‐values across methods.^9^ For the SC method, we use placebo tests for inference^2^, ^17^; for the GSC method, we use a bootstrap approach^41^; and for the DiD and IFE methods, we report *p*‐values based on cluster‐robust standard errors.

## 5 SIMULATION STUDY

We compare the methods in a Monte Carlo Simulation study where the true ATT is known and contrast the approaches according to mean bias (%) and RMSE. Building from the case study, we create 500 datasets of 111 units, of which 62 (49) were assigned to treatment (control) as in the case study^10^ and simulate data for up to 22 periods, with four of these assigned to be post‐treatment. The data generating process (DGP) includes one observed covariate (*X_it_*), 2‐way additive fixed effects (*μ_i_*_1_ and *λ*_1_ *_t_*), and a further two interacted factors and an additive treatment effect:

$$Y_{\mathrm{it}}=X_{\mathrm{it}}\beta +\mu_{i1}+\lambda_{1t}+\lambda_{2t}\mu_{i2}+\lambda_{3t}\mu_{i3}+D_{\mathrm{it}}\tau_{\mathrm{it}}+\varepsilon_{\mathrm{it}}$$

We draw $X_{i}$, $\mu_{i1}$, $\mu_{i2}$, and $\mu_{i3}$ from a standard multivariate normal distribution and $\lambda_{1t}$ from a uniform(0,5) distribution.^11^ To create a time‐varying $X_{\mathrm{it}}$, we then define $X_{\mathrm{it}}=0.5X_{i}+0.5*N\left(0,1\right)$. Here, $\varepsilon_{\mathrm{it}}$ is a standard normally distributed idiosyncratic error term. To introduce imbalance between the treated and control groups, the means of $\mu_{i1}$, $\mu_{i2}$, and $\mu_{i3}$ are set two standard deviations higher for the treated units than for the controls. In scenario A, we ensure the parallel trends assumption holds by setting $\lambda_{2t}=\lambda_{3t}=0$, so the DGP becomes a standard two‐way fixed effects model. In scenario B, we allow for monotonically increasing nonparallel trends by setting $\lambda_{2t}=0.2*t$ and $\lambda_{3t}=0.1*t$.

The performance of the SC method in scenario B may be negatively affected by our inclusion of time‐varying covariates ($X_{\mathrm{it}}$) since the SC weights are time‐invariant, and by the imbalance in **µ** leading to treated units that lie outside of the convex hull of the controls. Scenario C represents a setting without these specific challenges. Here, we use $X_{i}$ in place of $X_{\mathrm{it}}$ so that we have time‐invariant covariates, and to ensure that the average treated unit lies in the convex hull of the controls, for 25% of the control units we increase $\mu_{i2}$ and $\mu_{i3}$ by 4 standard deviations so that these unit's outcomes are likely to lie above those of the average treated unit, while the remaining 75% of controls tend to lie below. In scenario D, we include an additional postintervention shock, $\Delta \varepsilon_{\mathrm{it}}=2$, that only affects the treated group.

We consider scenarios (A1, B1, C1, and D1) where the treatment effect is homogenous ($\tau_{\mathrm{it}}$=1), and otherwise identical scenarios (A2, B2, C2 & D2) with a heterogeneous treatment effect, in which we define $\tau_{\mathrm{it}}=\left(1+\left(\mu_{i1}-2\right)\right)$.^12^ We then apply each method to estimate the average treatment effect for the treated group as a whole over the postintervention period. We consider the methods' performance across pretreatment periods of different lengths (6, 9, 12, and 18 periods). Finally, we assess the impact of imbalance in the numbers of treated (n = 10) vs control (n = 100) units (scenario E; Appendix S1).

## 6 RESULTS

### 6.1 Case study results

The estimated effects of the introduction of the BPT for hip fractures according to method are reported in Table 1. For both endpoints, the IFE method reports that the magnitude of the effect of BPT is larger than for the other methods. However, since differences in unobserved covariates, such as hospital quality, are likely to modify the effects of the policy, this may reflect bias due to heterogeneous treatment effects.

**Table 1:** Best Practices Tariffs case study results: ATT on process and outcome measures according to method

	Difference‐in‐differences	Synthetic controls	Interactive fixed effects	Generalized synthetic controls
Surgery within 48 h	0.0403	0.0482	0.0647	0.0590
	(*P* = .196)	(*P* = .250)	(*P* = .004)	(*P* = .004)
Dead within 30 d	−0.0080	−0.0051	−0.0123	−0.0062
	(*P* = .037)	(*P* = .560)	(*P* < .001)	(*P* = .308)

Note

For difference in differences, O'Neill et al^2^ report *p*‐values based on cluster robust standard errors. For the synthetic control method, *p*‐values are based on placebo tests as described in O'Neill et al^2^; for interactive fixed effects, we report *p*‐values based on cluster robust standard errors and the generalized synthetic control approach uses a bootstrap approach as described in Xu.^41^

The DiD, SC, and GSC methods provide similar point estimates. The *p*‐values do differ somewhat across the approaches, but the interpretation of these differences must recognize that the SC approach to inference differs to the other methods. The GSC method reports that the introduction of BPT increases the proportion of patients who have surgery within 48 hours, and suggests that the scheme leads to a reduction in mortality although this difference is not statistically significant.

### 6.2 Simulation results

**Figure 3:**
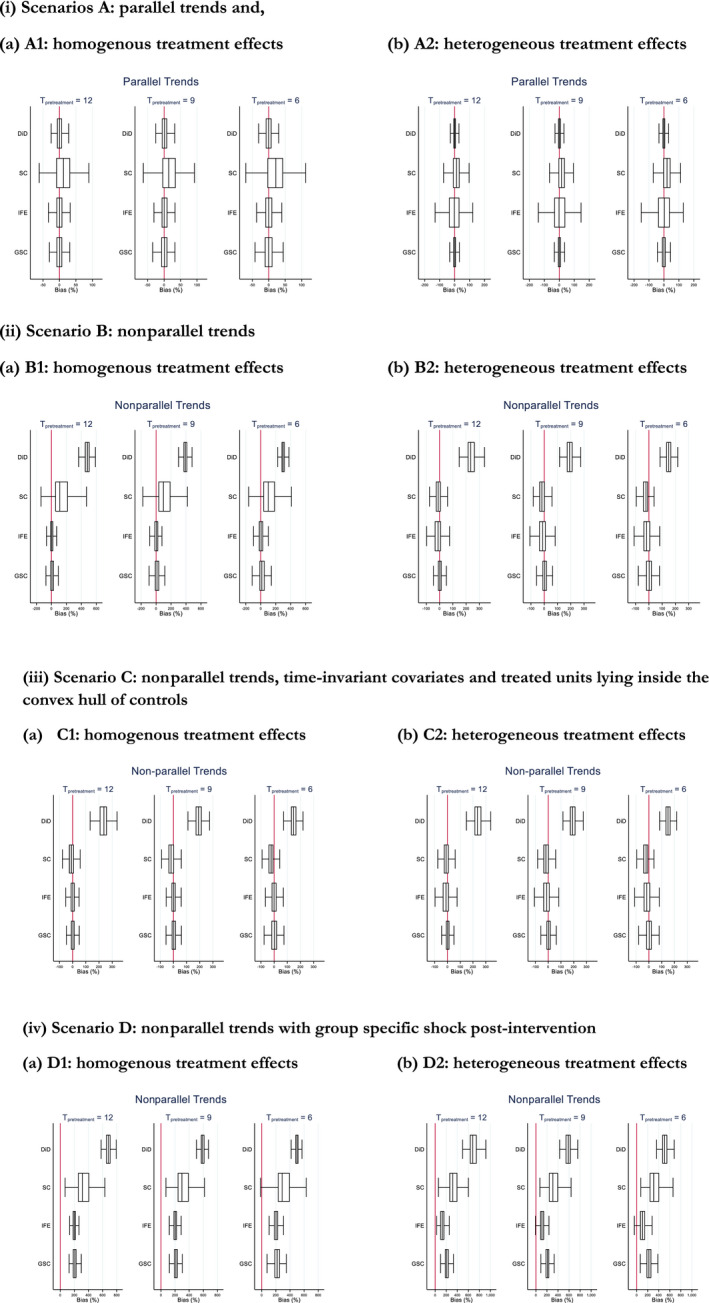
Boxplot of mean % bias in treatment effect estimates from Monte Carlo simulation. (i) Scenarios A: parallel trends, (ii) Scenario B: non‐parallel trends, (iii) Scenario C: non‐parallel trends, time‐invariant covariates and treated units lying inside the convex hull of controls, (iv) Scenario D: non‐parallel trends with group specific shock post‐intervention. *Note*: 500 simulations. *T*pretreatment is the number of pre‐treatment periods. Abbreviations: DiD, difference in differences; GSC, generalised synthetic control; IFE, interactive fixed effecs; SC= Synthetic control method [Color figure can be viewed at http://wileyonlinelibrary.com]

Figure 3 presents boxplots of the simulation estimates for each scenario by method, while Table 2 reports the corresponding mean bias (%) and root mean squared error (RMSE). We begin by considering the scenarios where effects are homogenous (scenario A1, B1, C1, and D1, panel (a) of Figure 3). As expected if the parallel trends assumption holds, DiD performs best (scenario A1), although IFE and GSC perform almost as well (Table 2, Figure 3(I)). By contrast, SC performs poorly, providing biased estimates attributable to the average treated unit tending to lie outside the convex hull of controls. Where the parallel trends assumption fails (scenario B1), DiD provides biased estimates, whereas IFE and GSC report minimal bias (Table 2, Figure 3(ii)). The SC method again provides biased estimates. In scenario C1, the performance of the SC method improves markedly (Table 2, Figure 3(iii)) since here the treated units tend to lie inside the convex hull of the controls. When a shock has a differential effect for the treated vs control group in the postintervention period (scenario D1), all methods provide biased estimates (Table 2, Figure 3(iv)).

**Table 2:** Monte Carlo simulation study results by method and scenario

Scenario	Root mean squared error							
	A1	A2	B1	B2	C1	C2	D1	D2
Parallel trends	Holds	Holds	Fails	Fails	Fails	Fails	Fails	Fails
Homogenous treatment effects	Yes	No	Yes	No	Yes	No	Yes	No
Time‐invariant covariates and treated units in convex hull	No	No	No	No	Yes	Yes	No	No
Group‐specific shock postintervention	No	No	No	No	No	No	Yes	Yes
18 pre‐treatment periods								
Difference‐in‐differences	0.01	0.01	43.74	43.74	10.78	10.78	74.25	74.25
Synthetic controls	0.09	0.09	4.46	4.46	0.06	0.06	14.88	14.88
Interactive fixed effects	0.01	0.36	0.06	1.88	0.02	0.11	4.37	6.43
Generalized synthetic controls	0.01	0.01	0.07	0.07	0.02	0.02	4.28	4.28
12 pre‐treatment periods								
Difference‐in‐differences	0.01	0.01	23.19	23.19	5.65	5.65	46.38	46.38
Synthetic controls	0.11	0.11	3.97	3.97	0.07	0.07	13.47	13.47
Interactive fixed effects	0.02	0.32	0.14	0.71	0.04	0.14	4.04	1.70
Generalized synthetic controls	0.02	0.02	0.11	0.11	0.03	0.03	4.30	4.30
9 pre‐treatment periods								
Difference‐in‐differences	0.01	0.01	15.34	15.34	3.78	3.78	34.93	34.93
Synthetic controls	0.13	0.13	3.80	3.80	0.10	0.10	12.98	12.98
Interactive fixed effects	0.02	0.34	0.17	0.98	0.05	0.17	4.19	1.52
Generalized synthetic controls	0.02	0.02	0.18	0.18	0.05	0.05	4.58	4.58
6 pre‐treatment periods								
Difference‐in‐differences	0.02	0.02	9.05	9.05	2.24	2.24	24.98	24.98
Synthetic controls	0.16	0.16	3.66	3.66	0.13	0.13	12.97	12.97
Interactive fixed effects	0.03	0.33	0.19	1.33	0.09	0.19	4.27	1.39
Generalized synthetic controls	0.03	0.03	0.34	0.34	0.10	0.10	5.00	5.00

In those scenarios with heterogeneous treatment effects (scenarios A2, B2, and C2, panel (b) of Figure 3), the GSC method continues to perform well, providing estimates with low bias and low RMSE (Table 2, Figure 3(i), (ii) (iii), (iv)). DiD, IFE, and SC all report biased estimates. For DiD, the bias is due to the failure of the parallel trends assumption. For the IFE model, the heterogeneous treatment effect biases the estimated values for $\lambda_{t}\mu_{i}$, which in turn biases the treatment‐free potential outcome and ultimately the ATT. For the SC method, the bias is attributable to poor overlap and is mitigated when the treated units lie in the convex hull of the controls (scenario C2). In scenario D2, all methods again report bias due to the postintervention shock.

## 7 DISCUSSION

This paper critically assesses two causal inference approaches, IFE and GSC methods, new to health policy evaluation, and contrasts them with DiD estimation and the SC method. The paper extends previous papers in the health policy and political science literatures^4^, ^5^, ^6^, ^7^, ^8^, ^9^, ^10^, ^11^, ^12^, ^13^, ^14^, ^15^, ^41^, ^74^ in contrasting IFE and GSC, but also approaches often considered in the HSR literature (DID and SC). Rather than focus solely on simple scenarios,^41^ the paper considers a range of settings relevant to the HSR context, including homogeneous and heterogeneous treatment effects, parallel tends and nonparallel tends, highly imbalanced numbers of treatment and control units, serial correlation, and idiosyncratic shocks. While our paper underscores the main finding from Xu's early simulation study,^41^ that GSC performs better than IFE when there is treatment effect heterogeneity, it offers a wider set of insights into the relative performance of GSC vs alternative methods in settings of direct relevance to the HSR context.

Our re‐evaluation of the BPT scheme exemplifies many critical issues faced in health policy evaluations. Here, there are multiple outcomes with the parallel trends assumption plausible for some but not others; the effects of the policy are anticipated to differ across hospitals; and data are only available for relatively few periods pre‐intervention. An attractive feature of the IFE and GSC methods is that they allow the analyst to adopt a consistent analytical approach across all outcomes, as their factor structure allows greater flexibility in controlling for unobserved confounders. However, the IFE estimator assumes homogenous treatment effects, which is unlikely in this study. Here, the GSC method is preferred in light of its robustness to the assumption of parallel/nonparallel trends and homogeneous/heterogeneous effects. It reported that BPT led to a large^13^ and statistically significant increase in the proportion of patients who had surgery within 48 hours of admission, together with a small, but not statistically significant, reduction in 30‐day mortality.

The simulation study found that the GSC approach performed better than the alternatives considered across a range of challenging settings typically faced in health economic and policy evaluations that use routine data, namely nonparallel trends, heterogeneous treatment effects, and few (6) pre‐intervention periods. However, when deciding which methods to apply to a particular setting, it is important to consider the underlying theory and requirements of the method. In particular, GSC and IFE approaches both require repeated observations of the same units over time (ie, panel data) and also require data for multiple pre‐intervention periods (one more than the specified number of interactive fixed effects to include).

Generalized synthetic control reports relatively precise estimates across all these challenging settings. We find the method performs well even if there is limited support for particular underlying causal assumptions (eg, parallel trends). In light of this, for the case study, which has some of these features, we emphasize the policy conclusions from the GSC approach, which is that the BPT intervention increased the probability of surgery within 48 hours, but does not lead to a change in 30‐day mortality. We also contribute to the growing literature that critically evaluates the SC method.^2^, ^26^ We extend O'Neill et al^2^ in recognizing that the SC method can perform badly if there is poor overlap in the pretreatment outcomes between the treated and control units, specifically when treated units lie outside the convex hull of the controls^17^, ^41^, ^14^. Conversely, we highlight that the SC method can perform well provided the treated observations do lie within the convex hull of the controls. Hence, future studies should consider carefully whether their evaluations have these features before opting for SC as an alternative for DiD estimation.

This paper has the following limitations. First, each of the methods considered assumes that idiosyncratic shocks postintervention have the same expected effect on outcomes for the treated and control groups. Similarly, while any of these approaches can incorporate individual‐level baseline information, for example, on patient case mix, by “risk adjusting” outcomes, unobserved compositional changes in the postintervention period may be wrongly attributed to the effect of the intervention. Second, to aid transparency, the Monte Carlo simulation study had a relatively simple DGP and assumed the IFE models including the one underlying the GSC method were correctly specified. A natural next step would be to contrast the IFE and GSC approaches to other relatively untested methods from the general causal inference literature.^29^, ^31^, ^32^, ^33^ Third, in empirical studies the methods would ideally be contrasted by applying the same randomization inference procedure. The Conley‐Taber randomization inference procedure has been recommended for this purpose, but requires the same number of observations across the treated and control groups.^63^

The findings from this paper and ongoing methods development more widely highlight two complementary areas for further research. First, a number of extensions to DiD have been proposed to increase the validity of DiD‐type estimators including: allowing for unit‐specific trends,^64^, ^65^ combining matching with DiD,^66^ and combining instrumental variables (IV) approaches with DiD.^67^ While combining IV with DiD would allow for unobserved confounding, the population this estimate relates to (compliers) may not be of policy relevance.

Second, the limitations of the originally proposed SC method^16^, ^17^ have led to recent modifications. The augmented SC approach^71^ addresses the bias due to non‐exact balance on pretreatment outcomes. The imperfect SC^35^ reduces the sensitivity of estimates to idiosyncratic errors by applying SC to predicted rather than actual outcomes. A number of approaches relax the overlap requirement by allowing for negative weights.^29^, ^35^, ^71^ Extensions of the SC method using machine learning methods such as ridge regression^71^ and the matrix completion approach^31^ appear promising. Inference for SC type methods is an area of active research, with several authors proposing extensions to the originally proposed placebo tests.^29^, ^32^, ^70^ Future work is required that considers the relative performance of these methods and reports the coverage of alternative inferential procedures.

## ENDNOTES

1 A recent working paper by Schmidt et al^69^ uses the GSC method to assess whether insurance coverage of medical treatments with high out‐of‐pocket costs affects patients' utilization.

2 See McDonald et al^42^ and Kristensen et al 68 for further details.

3 Here, participation status is defined according to whether the hospital trust had reported receiving any BPT payments for hip fractures in 2010/11.^42^

4 The test for parallel trends is described in Appendix C of O'Neill et al^2^

5 The general framework in equation 1 also nests unit‐specific linear trends,^64^, ^65^ which would be obtained if we specify $\mu_{i}=\left[1,\mu_{i},\mu_{i}\right]$ and $\lambda_{t}=\left[\lambda_{t},t,1\right]$.

6 Under staggered adoption, and heterogeneous effects, extra care must be taken to identify the effect being estimated.^72^, ^73^

7 Gobillon and Magnac^30^ suggest a similar approach that uses an expectation maximization approach.

8 Sample code to estimate all of the methods is available from the authors on request.

9 For instance, placebo tests capture whether the estimated effect for the treated group (or unit) is large relative to the effect that would have been estimated for a treatment group (or unit) chosen at random.^17^ This contrasts with the more common random sampling perspective underlying standard errors for regression models.^28^

10 Results were similar when 10 treated units and 100 control units were used instead.

11 We allow for correlation between $X_{\mathrm{it}}$, $\mu_{i1}$, $\mu_{i2}$, and $\mu_{i3}$ with the correlation matrix, C = (1, 0.5, 0.5, 0.3 \ 0.5, 1, 0.5, 0.3 \ 0.5, 0.5, 1, 0.5 \ 0.3, 0.3, 0.5, 1).

12 Note that since E(μ_i1_ |D_it_ = 1)=2 here, the true ATT is 1 in all scenarios.

13 The average rate of surgery within 48 hours was 58.3%.

14 Where the treated unit's outcomes are very different to those of the controls, the most similar control will receive a weight of 1 and be used as the counterfactual for the treated unit, even though it may be very dissimilar.
