# Supplementary material for: A comparison of methods for health policy evaluation with controlled pre‐post designs
Source: Health Serv Res. 2020 Feb 12;55(2):328–38. doi: 10.1111/1475-6773.13274 (PMC7080394; doi:10.1111/1475-6773.13274)
Supplement: Supplementary file 2 [file HESR-55-328-s002.docx]

**Online Appendix:**

**Table A1: Monte Carlo simulation study results by method for scenario E which includes many (100) controls and few (10) treated units.**

|  | **RMSE** | |
| --- | --- | --- |
| **Scenario:** | **E1** | **E2** |
| **Parallel trends:** | **Fails** | **Fails** |
| **Homogenous treatment effects:** | **Yes** | **No** |
| **Time-invariant covariates & treated units in convex hull** | **No** | **No** |
| **Few Treated units (10) relative to Controls (100)** | **Yes** | **Yes** |
| **Group specific shock post intervention** | **No** | **No** |
|  |  |  |
| ***18 pre-treatment periods*** |  |  |
| Difference-in-Differences | 44.58 | 44.58 |
| Synthetic Controls | 3.32 | 3.32 |
| Interactive Fixed Effects | 0.11 | 0.15 |
| Generalised Synthetic Controls | 0.11 | 0.11 |
|  |  |  |
| ***12 pre-treatment periods*** |  |  |
| Difference-in-Differences | 23.19 | 23.19 |
| Synthetic Controls | 3.14 | 3.14 |
| Interactive Fixed Effects | 0.17 | 0.24 |
| Generalised Synthetic Controls | 0.15 | 0.15 |
|  |  |  |
| ***9 pre-treatment periods*** |  |  |
| Difference-in-Differences | 15.41 | 15.41 |
| Synthetic Controls | 3.13 | 3.13 |
| Interactive Fixed Effects | 0.24 | 0.36 |
| Generalised Synthetic Controls | 0.25 | 0.25 |
|  |  |  |
| ***6 pre-treatment periods*** |  |  |
| Difference-in-Differences | 9.23 | 9.22 |
| Synthetic Controls | 2.59 | 2.59 |
| Interactive Fixed Effects | 0.37 | 0.48 |
| Generalised Synthetic Controls | 0.49 | 0.49 |
|  |  |  |

**Details on computation time.**

*In our case study, for a single outcome, GSC took 200 seconds on an Intel Core i7-6700 CPU @ 3.40GHz with32gb of RAM to estimate the effect, using cross-validation to determine the most appropriate number of factors (from 0 to 5) and providing confidence intervals based on 500 bootstraps. IFE took 7 seconds, DiD took 2 seconds and SC took 259 seconds, including the placebo test. Given space constraint, however, we did not think this was of sufficient priority to include in the paper.*
